# The role of telemedicine during the COVID-19 epidemic in China—experience from Shandong province

**DOI:** 10.1186/s13054-020-02884-9

**Published:** 2020-04-28

**Authors:** Xuan Song, Xinyan Liu, Chunting Wang

**Affiliations:** 1 ICU, Liaocheng Cardiac Hospital Affiliated to Shandong First Medical University, Liaocheng, Shandong China; 2 Division of Pulmonary and Critical Care Medicine, Department of Medicine, Mayo Clinic, Rochester, MN USA; 3 ICU, DongE Hospital Affiliated to Shandong First Medical University, Liaocheng, Shandong China; 4 ICU, Shandong Provincial Hospital Affiliated to Shandong First Medical University, 16766 Jingshi Road, Jinan, 250014 Shandong China

**Keywords:** Coronavirus, COVID-19, Pandemics, Coronavirus infections, Remote consultation, Severe acute respiratory syndrome coronavirus 2

In December 2019, an outbreak of 2019-coronavirus (COVID-19) caused a substantial public health crisis in Wuhan, China, growing into a global pandemic [1]. With a population of > 100 million, Shandong province is an enormous, economically developed province with frequent population movement. It is in East China and close to Henan province (the third largest population in China), South Korea, and Japan. Therefore, preventing an epidemic was critical in Shandong province.

As of March 26, 2020, 759 confirmed cases, including 71 critically ill patients and seven deaths, occurred in Shandong Providence, with a reported mortality rate of 0.92%, which was lower than the national mortality rate of 4.01%. The lower incidence and death rates potentially indicate the preventive strategies in Shandong providence were effective. The Shandong provincial government and the Shandong Health Committee responded quickly to the epidemic and established “Anti-epidemic Expert Group” to formulate diagnosis, treatment, quarantine, and reporting protocols. In the management of COVID-19 patients, the expert group comprehensively utilized all professional platforms, including telemedicine to connect patients, experts, and information. Additionally, telemedicine provided prevention and treatment guidance, training, communication, and remote consulting for the community residents and medical staff. The Anti-epidemic Expert Group found the telemedicine platform to play a considerable role in controlling the COVID-19 epidemic in the Shandong province (Fig. 1). Here, we describe the experiment and the benefits of using telemedicine for the prevention of COVID-19.

**Fig. 1:**
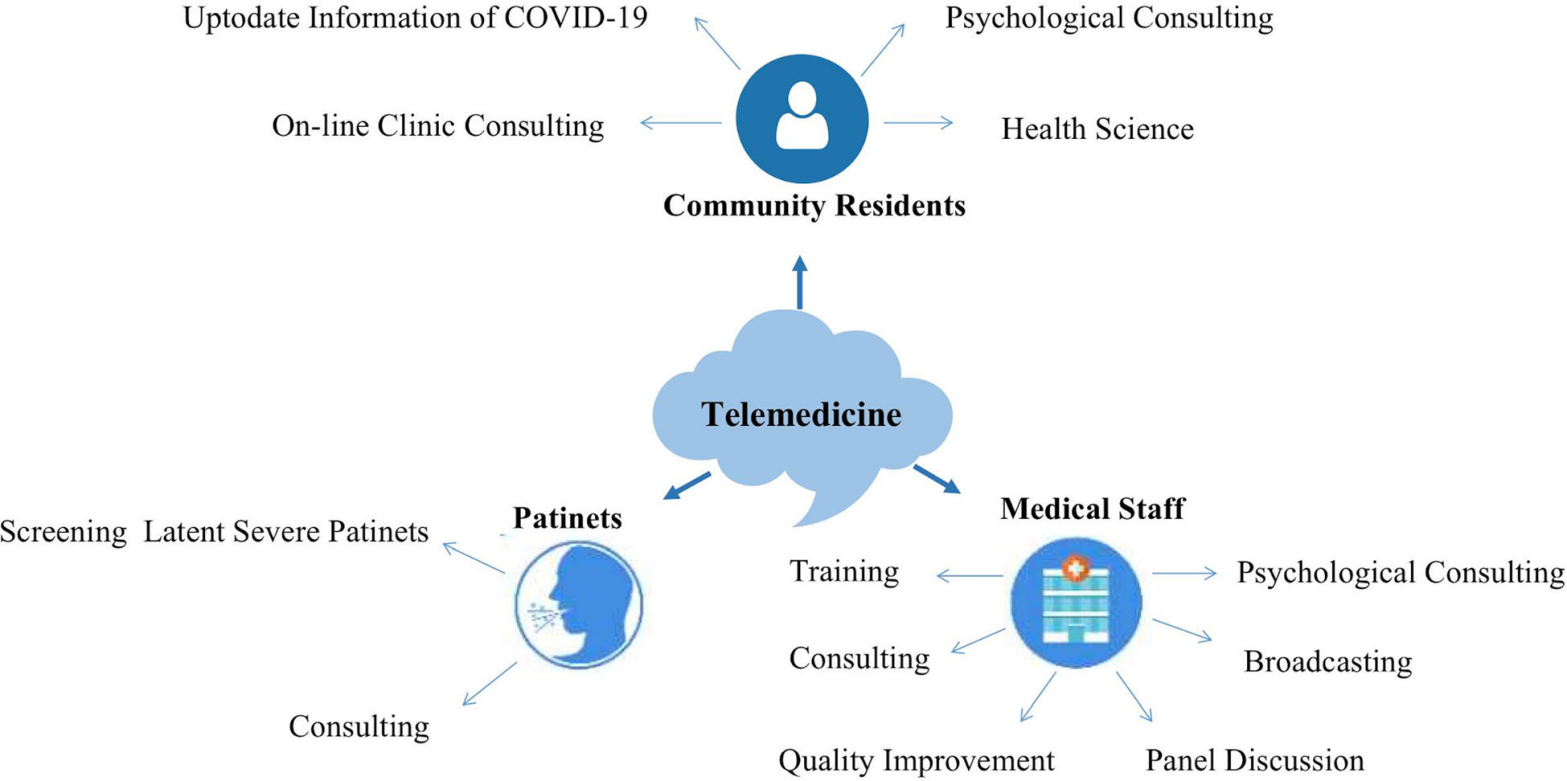
Telemedicine platform function

## Community residents

Telemedicine platform included a COVID-19 informational page, which updated the latest information in real time, including instructions for quarantine processes at home, personal protection applications, and time for seeking medical attention. On January 24, 2019, remote education for vulnerable individuals, including pregnant women, was initiated. During this epidemic, only one pregnant woman was severely ill in Shandong province with very favorable outcomes.

The telemedicine platform also included an online consulting clinic, where experts were available 24 h/day. Experts could conduct preliminary screenings through remote consultation, which avoided the risk of cross infection in the hospitals and relieved pressure away from designated hospitals. Community residents and providers felt it created favorable support for early detection, diagnosis, and prevention.

## Patients

**Fig. 2:**
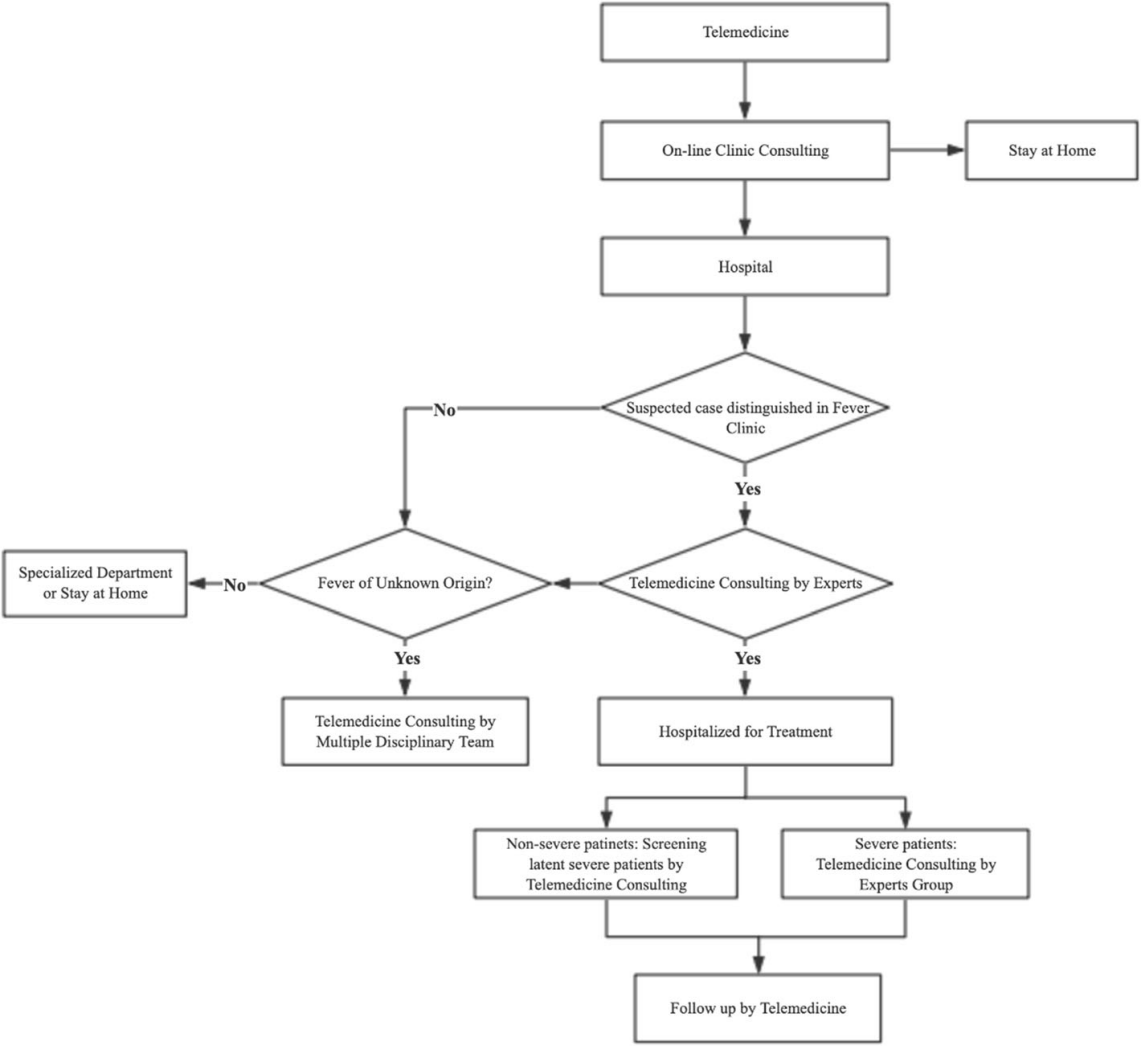
COVID-19 patient management protocol by telemedicine

Community residents with symptoms could consult through online clinic consultation services. Experts conducted preliminary screenings of patients online and gave suggestions to continue to stay home or to go to the hospital. After arrival at the hospital fever clinic, clinicians determined whether patients were suspected for COVID-19. Following this initial triage, an expert conducted a remote consultation for further determination of the risk of COVID-19. Non-severe cases were assessed according to the following criteria for the severity of latent instances: (1) the National Early Warning Score (NEWS) score and (2) age > 65 years [2, 3]. These cases were reported to the expert group via telemedicine every day, thereby implementing early preventive interventions for this group of patients. For cases with severe illness, remote consultation could be arranged at any time via telemedicine. Patients discharged from the hospital were followed up via telemedicine. Telemedicine seamlessly monitored patients from initial triage through to post-discharge, while also collecting patient data to be incorporated into a future database (Fig. 2). During the epidemic period, 582 cases were consulted in Shandong province and 15 in Hubei province. Telemedicine consulting saved time and cost, while decreasing the risk of infection distribution by avoiding close contacts with patients with COVID-19.

## Medical staff

Due to the heterogenous distribution of medical resources across Shandong, the level of medical treatment availability varied. Many regions do not have intensive care units (ICU) or adequate ICU medical staff. Additionally, most designated COVID-19 hospitals focus on infectious disease but lack experience in the management of critically ill patients. Experts in pulmonary and intensive care medicine and infectious diseases have different experiences and resources with regard to patient management, including oxygen therapy, intubation timing, and application of therapeutic agents like corticosteroids. Homogenizing and refining management of COVID-19 patients is vital to improving patient outcomes.

The Shandong Anti-Epidemic Expert Group utilized the “telemedicine” platform to carry out epidemic control activities in Shandong and Hubei provinces. Also, Shandong province supported medical teams, not only in China but also around the globe. On January 23, 2020, telemedicine shared personal protection videos and conducted remote training for healthcare providers in Shandong province. Concurrently, 50 training and panel discussions were held nationwide by telemedicine. Expert panels from intensive care, respiratory, infectious disease, and traditional Chinese medicine backgrounds were invited to connect with Hubei province colleagues. Topics included diagnosis, respiratory support, circulation, immunization, extracorporeal membrane oxygenation (ECMO) treatment, case discussion, personnel protection, psychological counseling, and management of critically ill patients with COVID-19. More than 500,000 individuals participated in these video conferences.

The World Health Organization (WHO) recently declared COVID-19 a pandemic. The Shandong province expert group invited Chinese topic authorities to use the “Cloud ICU” platform to share their experience managing critically ill patients with COVID-19 to help mitigate the global spread of COVID-19 using telemedicine. The platform is equipped with an anti-epidemic diary and telephone hotline to help reduce medical staff burnout, which has given healthcare providers a chance to communicate with each other, develop a sense of belonging to the community, and a secure feeling, allowing them to remain vigilant in fighting this disease.

COVID-19 is an entirely new disease with emerging conflicting information in the media and internet [4]. This information overload coupled with tremendous patient workload makes healthcare providers prone to burnout and depression. Telemedicine is a professional and reliable information platform that delineates factual information, allowing medical staff to obtain reliable information in real time with appropriate authentication. Additionally, medical staff can communicate with colleagues, listen to lectures, apply for consultations, etc. This platform is considered safe, trustworthy, convenient, and time-, labor-, and cost-saving.

Telemedicine activities avoid close contact and decrease the latent COVID-19 infection chance; therefore, in this peculiar period, telemedicine plays a huge role. Based on our positive experience using telemedicine, we suggest establishing similar professional telemedicine platforms, using remote technologies to integrate resources, share information, and support healthcare providers. We believe if everyone works together, an ultimate victory over this pandemic is achievable.

## Data Availability

Not applicable.

## Abbreviations

COVID-19: Coronavirus 2019
ECMO: Extracorporeal membrane oxygenation
ICU: Intensive care unit
NEWS: National Early Warning Score
WHO: World Health Organization
